# Status and trends in the international wildlife trade in Chameleons with a focus on Tanzania

**DOI:** 10.1371/journal.pone.0300371

**Published:** 2024-05-16

**Authors:** Maxim Conrad Isaac, Neil D. Burgess, Oliver J. S. Tallowin, Alyson T. Pavitt, Reuben M. J. Kadigi, Claire Ract

**Affiliations:** 1 CMEC, within GLOBE Institute, University of Copenhagen, Copenhagen, Denmark; 2 UN Environment Programme World Conservation Monitoring Centre (UNEP-WCMC), Cambridge, United Kingdom; 3 IUCN, Cambridge, United Kingdom; 4 Department of Trade and Investment, College of Economics and Business Studies, Sokoine University of Agriculture, Morogoro, Tanzania; Eduardo Mondlane University: Universidade Eduardo Mondlane, MOZAMBIQUE

## Abstract

Chameleons (family Chamaeleonidae) are a distinctive group of reptiles, mainly found in Africa, which have high local endemism and face significant threats from the international wildlife trade. We review the scale and structure of international chameleon trade, with a focus on collection in and exports from Tanzania; a hotspot of chameleon diversity. Analysis used data from the CITES Trade Database 2000–2019, combined with assessment of online trade, and on-the-ground surveys in Tanzania in 2019. Between 2000 and 2019, 1,128,776 live chameleons from 108 species were reported as exported globally, with 193,093 of these (from 32 species) exported by Tanzania. Both global and Tanzanian chameleon exports declined across the study period, driven by decreased trade in generalist genera. Whilst the proportion of captive-bred individuals increased across time for the generalist taxa, the majority of range-restricted taxa in trade remained largely wild-sourced. For Tanzanian exports, 41% of chameleons were from one of the 23 endemic species, and 10 of the 12 Tanzanian endemic species in trade are categorised as threatened with extinction by IUCN. In terms of online trade, of the 42 Tanzanian species assessed, there was evidence of online sale for 83.3% species, and 69% were actively for sale with prices listed. Prices were on average highest for *Trioceros* species, followed by *Kinyongia*, *Rieppeleon*, *Rhampholeon*, and *Chameleo*. Field work in Tanzania provided evidence that the historic harvest of endemic chameleon species has been higher than the quantities of these species reported as exported by Tanzania in their annual trade reports to CITES. However, we found no field evidence for trade in 2020 and 2021, in line with Tanzanian regulations that applied a blanket ban on all exports of live wild animals. Literature evidence, however, suggests that illegal trade continued to Europe from seizures of Tanzanian chameleon species in Austria in 2021.

## 1. Introduction

Chameleons (family: Chamaeleonidae) occur throughout continental Africa, Madagascar, southern Europe, the Arabian Peninsula and the Indian subcontinent [1], with hotspots of species richness and endemism in Madagascar, and South and East Africa [2, 3]. The United Republic of Tanzania (hereafter referred to as Tanzania) is particularly important on mainland Africa [4], supporting about 20% of all known species (41 of 222 species; [5]). Recent research suggests that Tanzanian species diversity may be even higher, particularly in the Eastern Arc Mountains [6].

Of the 205 chameleon species with IUCN Red List assessments (version 2022–2), 37% (78) are classified as globally threatened (10 Critically Endangered [CR], 43 Endangered [EN] and 25 Vulnerable [VU]); the remaining species were classified as Near Threatened (37), Data Deficient (10) or Least Concern (80) [7]. Multiple pressures threaten chameleons, but a major threat is collection for the international pet trade which exacerbates the threats posed by habitat clearance and fragmentation, and climate change, which are reducing the area of suitable mountain forest habitat for many narrow ranging species [8–10].

Chameleons are popular in the reptile pet trade, with wild populations of enigmatic and rare species at the greatest risk of unsustainable over-collection [1, 11]. Trade in 202 chameleon species is currently regulated through the Convention on International Trade in Endangered Species of Wild Fauna and Flora (CITES): one species (*Brookesia perarmata*) is listed in CITES Appendix I, which affords the highest level of protection, and the remaining 201 species are listed in CITES Appendix II. However, under-recorded illegal and domestic trade, and a poor understanding of the life history of most species, means the overall impact of harvest and trade on these species is poorly understood [12–14].

In the past reptiles would have been traded at reptile markets, however, the internet has helped broaden the interest in such species and increased the trade network [15]. Increased interest in exotic pets and better access to wild species has led to greater demand in colourful, enigmatic species [16], and long-term cultural preferences for the most attractive species has led them to be among the most threatened species today [17]. Collectors now have access to papers describing species localities and geotagged photographs enabling them to target the collection of specific rare, endemic or threatened species [11].

The global trade in CITES-listed chameleons was previously assessed in 2004 [1], however trade in range restricted and endemic chameleon species, as well as the relationship between on-the-ground harvesting and recorded exports, remain under-examined. National trade has previously been assessed for Madagascar [1, 9], but detailed assessment of harvest and international trade in other chameleon hotspots is limited. Furthermore, studies of reptile trade provide us with general trends amongst all lizards but lack the level of detail needed to understand the mechanics of chameleon trade [18] only identifying the impacts on a handful of generalist species [14].

Despite the diversity of chameleons in Tanzania, little is known about trade from this biodiversity hotspot, although previous work has raised concerns about the sustainability of the harvest of chameleons from Tanzania [9, 10]. A blanket ban on the export of all live wild-sourced animals from Tanzania has also been in place since 2016, with no national CITES export quotas published since 2017 as a result. The trade in two chameleon species is further regulated under CITES, with trade suspensions in place for *Kinyongia fischeri* and *K*. *tavetana* since 2016, prohibiting the export of both captive- and wild-sourced specimens from Tanzania.

In this paper we explore the international trade in CITES-listed chameleons, with a focus on species exported from Tanzania. We address three main objectives: [1] provide an overview of the international trade in CITES-listed chameleons (family: Chamaeleonidae) from Tanzania 2000–2019, in the context of global chameleon trade; [2] assess similarities and differences between the international trade in chameleons from Tanzania as reported to CITES, and data collected from online marketplaces and on-the-ground surveys in Tanzania; and [3] build a detailed picture of the structure of chameleon collection and trade system within Tanzania.

## 2. Methods

### 2.1. Taxonomy

We used “The Reptile Database” for this study’s core taxonomy. Data from the IUCN Red List (e.g. estimated area of occupancy (S2 Table in S1 Annex) [5]) and CITES Trade Database were mapped onto this central taxonomy by accepted name.

### 2.2. CITES trade database

The CITES Trade Database (trade.cites.org) provides insights into the international trade in chameleons (family: Chamaeleonidae). The database includes international trade records in CITES-listed species as reported by the 184 CITES Parties (183 signatory countries and the European Union) in their official annual reports to the Convention. The database is maintained by the UN Environment Programme World Conservation Monitoring Centre (UNEP-WCMC) on behalf of the CITES Secretariat.

Data for the 20-year time period 2000–2019 were extracted from the CITES Trade Database as a comparative tabulation report on 11^th^ of June 2021, with each record containing data on the importing country/territory, exporting country/territory, reporter type (i.e. was the trade reported by the exporting or importing Party), quantity and unit of measure, year, taxon, CITES listing, trade term (type of product, e.g. live specimens, skins), the purpose of the trade (e.g. commercial, scientific) and the source (e.g. wild, captive bred, ranched). Data were standardized to current CITES trade terms and standard units of measure to ensure trade was comparable. Exporter-reported data was used for analysis of global trade, as countries are not required under CITES to issue import permits for Appendix II species (although some, such as the EU, do issue import permits under stricter domestic measures), consequently exporter-reported trade is likely to be more complete for purposes of this analysis [19, 20].

### 2.3. Data preparation

Only trade reported by number of specimens (i.e. unit code ‘blank’) in trade terms that could be equated to one individual (trade term codes: ‘live’, ‘bodies’, ‘skeletons’ and ‘skulls’) were included in this analysis; trade reported as other units of measures (e.g. weight), or in trade terms that could not be equated to one individual (e.g. ‘claws’, ‘eggs’, ‘skin pieces’, or ‘derivatives’) were excluded.

We excluded records with ‘purpose’ codes other than ‘T’ for commercial trade, this was because preliminary analysis (see S1 Fig in S1 Annex) revealed trade for other purposes to account for less than 1% of all chameleon trade and focusing our analysis of global trade and allows for a clearer understanding of the commercial impact of the trade.

Our study wished to analyse the variable sources of chameleons (wild, captive bred, ranched etc) so all source codes were selected initially, before reducing the scope to focus on wild sourced trade (source codes ‘W’, ‘U’ and ‘blank’) similarly our study focused on CITES listed species to understand the impacts trade regulations have on chameleons. (Information on current and historical status of a species CITES listing can be retrieved from Species+ (speciesplus.net)) As such only records classified as Appendix I, II or III were selected for analysis.

### 2.4. Data analysis

For analysis of global trade, direct (i.e. origin reported as blank) exporter-reported trade from all exporting countries and territories was included; indirect trade (i.e. re-exports) was excluded to avoid double-counting. Maps of trade routes were created using Tableau and the data for these was prepared using Alteryx.

To analyse trends in the CITES trade data at the global level, the data were aggregated in several ways: (a) by year to analyse global trade trends over time, (b) by genus to explore the chameleon genera most in trade, and (c) by exporting country/territory (summed for the years 2000–2016 only to account for Tanzania’s trade ban since 2017) to identify the top ten global exporters by quantity. For the top ten exporting country/territories, the number of native vs non-native species was then calculated based on distributions listed in the Reptile Database (reptile-database.reptarium.cz).

Trade diversity (i.e. the number of species in trade) was calculated for each exporting country 2000–2019 and compared to the total number of chameleon species considered native to that country. The number of species in trade was also disaggregated by year to explore temporal trends and the relationship between trade diversity and quantities in trade.

For our analysis of global chameleon trade by genus the data was filtered by genus and included all genera listed in the database that met the requirements.

For analysis by exporter the data was sorted by total exports by nation during the period 2000–2019 and the ten most prolific exporters (defined by total exporter reported exports over the study period) were examined.

For analysis of wild and captive sourcing in global chameleon trade the data was analysed by source codes, sourcing analysis was conducted for the top ten exporting nations in two categories, range countries and non-range countries for chameleons, these were determined using the Reptile Database (reptile-database.reptarium.cz).

For analysis of diversity of species in trade the trade quantities were ordered by taxon and then using the COUNTIF function were used to create a binary count of whether each species in trade during the study period was in trade each year. This generated a species count for each year, this was then compared with export numbers from all exporting nations and additionally the number of chameleon species present in each nation (again found using the Reptile Database).

### 2.5. Online surveys of trade in Tanzanian Chameleons

We searched online marketplaces using English language search terms aiming to discover which species are sold on the open market, which species are in demand, and, where possible, the approximate price range for different species. Common practice in web-surveying for wildlife products [21] was observed. We also followed ethical guidelines for surveying social media sites and forums [22, 23]. No member names were collected, we did not visit personal pages, did not use automated web scrapers, and no other personal information was collected about members of these online communities.

In total, 14 conventional pet store websites, two online forums, two online ‘classified ad’ pages and four social media pages and seven closed social media ‘groups’ were surveyed. Keyword searches using accepted scientific names (according to the Reptile Database) for all chameleon species present in Tanzania were performed to identify the suitability of these websites for surveying. This was done by recording the websites evidencing sale of chameleons on the first page of results obtained by searching “[species name] for sale” on google.com, the 10 most frequently appearing pages were selected to survey (S4a Table in S1 Annex). Social media pages requests for access were submitted to closed groups of chameleon enthusiasts and public pages for reptile sellers on “Facebook” were scanned for evidence of direct selling on these pages. Additionally, “Facebook marketplace” was searched, using the aforementioned cross-section of common English and scientific names. The price, availability status and sale location were recorded from these searches, and a mean price was taken for each species for further analysis.

### 2.6. Field research in Tanzania

Field research was conducted in Tanzania 23^rd^ of July– 24^th^ of September 2019 aiming to establish sources of animals sold on the international export market, the scale of harvesting of range restricted species and the characteristics that make species more desirable. Semi-structured interviews with local villagers were conducted at various locations in the Eastern Arc Mountains (EAM) and used to gather information on trade systems, these interviews were structured around 11 questions (S5 Table in S1 Annex) designed to establish the scale of harvesting, the demographic of people involved, structure of the trade and the species targeted whilst leaving scope for other commentary interviewees wished to provide. The EAM were selected as a focal study area due to their high levels of endemism for chameleons [2, 6, 8].

**Fig 1:**
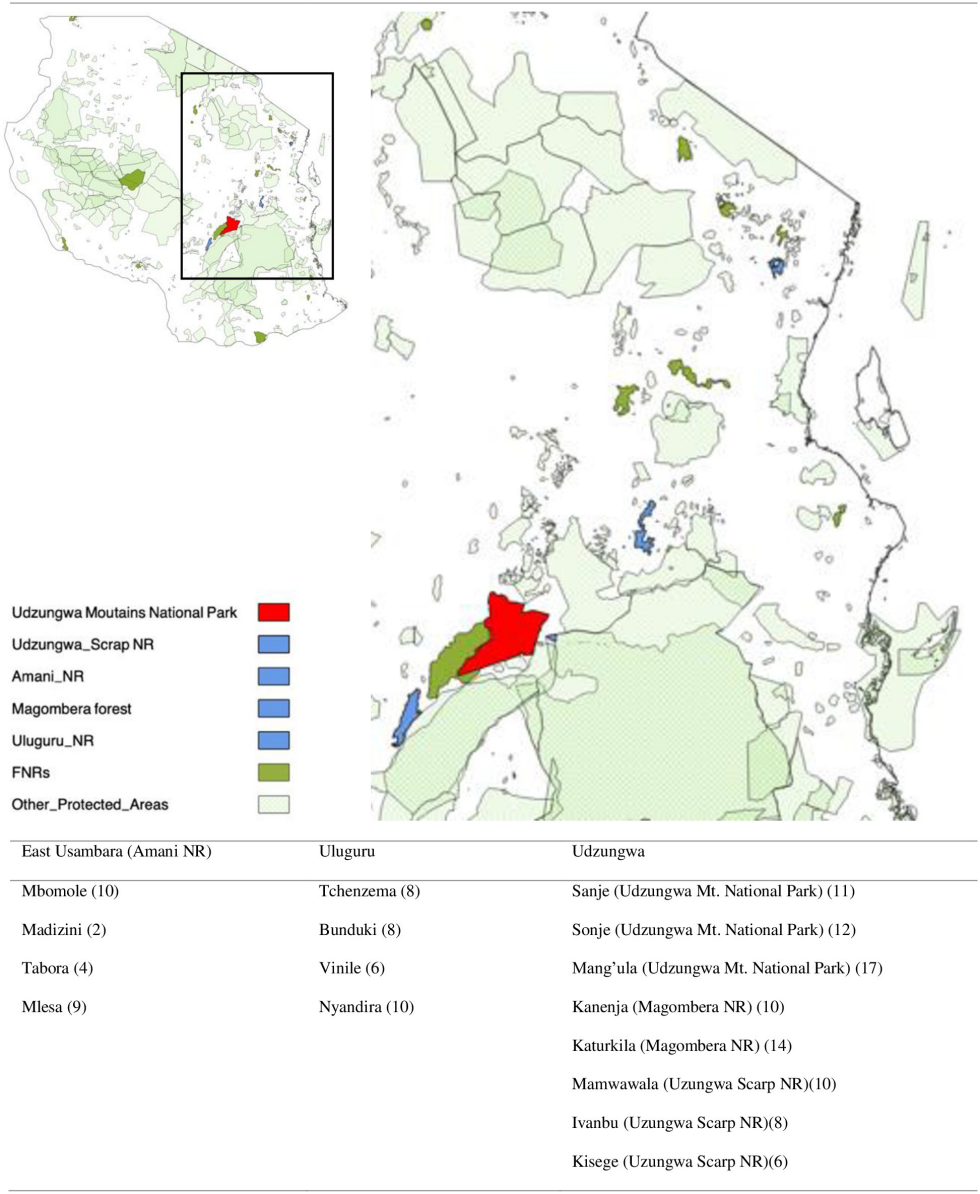
Location of protected areas surveyed across the Eastern Arc Mountain chain, with villages and number of people surveyed around each reserve indicated below. A) Reserve network of Tanzania with surveyed Nature Reserves (blue) and National Parks (red) highlighted, with non-surveyed reserves in green. B) Reptile species richness across Tanzania showing the location pf the Eastern Arc Mountains ecoregion (from Meng et al., 2016). C) Villages surveyed in each mountain block (reserve name and number of respondents in brackets).

The field locations in the EAM were villages surrounding forest nature reserves and national parks in three mountain blocks: East Usambara in the north of Tanzania; Uluguru in Morogoro Region; and Udzungwa in the southern end of the EAM chain. These three locations were chosen due to; a) their proximity to major population centres/potential markets/major highways; b) known presence of endemic chameleon species; c) presence of a national park or nature reserve conserving forest areas; and d) known past involvement in the chameleon trade (Fig 1).

Semi structured interviews were conducted with residents in the study villages. Interviewees were selected based on the expertise of local guides/foresters. People were interviewed both individually and sometimes in small groups where individual interviews were not possible. A translator asked the questions when villagers did not speak English.

In the Uluguru and East Usambara mountains, collectors’ preference for different types of chameleon species was investigated by asking villagers what they target in collection. Species-specific popularity can be inferred from preferred physical characteristics, namely how many horns the chameleons possess (none, one, two, three)—or by body size, namely the giant chameleon *Trioceros melleri* [4]. Grouping species by number of horns was not always possible for sexually dimorphic species where females and males differed in the number of horns (see Table 1). In some cases the classification by horn number narrows identification to one possible species (*K*. *oxyrhina* and *K*. *uluguruensis* in the Uluguru, *T*. *deremensis* & *K*. *matschiei* in the Usambara), but in other cases there are several possible candidates, particularly for specimens with no horns [4].

**Table 1:** Trade and prices of Tanzanian Chameleons in trade since 2000 (last trade registered 2016 due to blanket ban (1USD = 2299 Tanzania Shillings [01/08/2019]).

	One horn	Two horns	Three horns	No horn	Giant
Average price paid (range Uluguru–Usambara)	955–3750 tsh / 0.42–1.63 USD	1402–3375 tsh/ 0.61–1.47 USD	1000–3875 tsh/ 0.43–1.68 USD	900–3125 tsh/ 0.39–1.36 USD	1204–4475 tsh/ 0.52–1.95 USD
Annual number of live, wild-sourced chameleons reported by survey respondents as harvested from Uluguru and Usambara	5560 individuals/year	5776	5219	4410	306
Mean annual number of live chameleons reported as exported by Tanzania in their annual reports to CITES 2000–2016*	12 individuals/year	0	774	2693	1850
Tanzanian endemic chameleon species (mean annual number of live individuals reported as exported by Tanzania in their annual reports to CITES 2000–2016* in parentheses)	*K*. *oxyrhina* (3)	*K*. *uluguruensis* (0)	*T*. *deremensis* (411)	*Rieppeleon brachyurus* (0) *Chamaeleo dilepis* (2693)	*T*. *melleri* (1850)
	*K*. *tenuis* (4)	*K*. *matschiei* (0)			
	*Rhampheleon*	*K*. *vosseleri* (0)	*T*. *werneri* (362)		
	*spinosus* (5)	*K*. *multiturbuculata* (0)			
	*Rhampheleon temporalis* (0)				

In addition, key informant interviews were conducted with people in Tanzania involved in fields with relevance to the wildlife trade. These included conservationists and academics, Non-Governmental organisations (WWF & TRAFFIC Tanzania, UNDP Tanzania) and government authorities (Tanzania Wildlife Management Authority—TAWA, Tanzania Wildlife Research Institute—TAWIRI, Tanzania Forest Services agency).

Patterns and trends in the collection of chameleons obtained from the field surveys, including specifically targeted species, were considered in light of the official CITES export data reported by Tanzania and accessed via in the CITES Trade Database. Details of any changes in export policies by the Tanzania government since 2019 were provided by one of the authors (RMJK).

## 3. Results

### 3.1. Global Chameleon trade

Our analysis of the global trade in CITES-listed chameleons shows an overall significant (R^2^ = 0.35, P<0.001) decline in reported international trade during the study period of 2000–2019. Over time there has been a significant (R^2^ = 0.75, P<0.001) decrease in reported trade in wild sourced chameleons, an increase in captive bred chameleons, and a slight overall decline in ranched specimens globally (S2b Fig in S1 Annex). Chameleon exports were overwhelmingly for commercial purposes (see S1 Fig in S1 Annex). Most ranched and captive bred species were in the genus *Chameleo*, whilst trade in range-restricted genera were predominantly collected from the wild (S2a Fig in S1 Annex); the diversity of species being captive bred and ranched did not change significantly over the study period (S2b Fig in S1 Annex).

**Fig 2:**
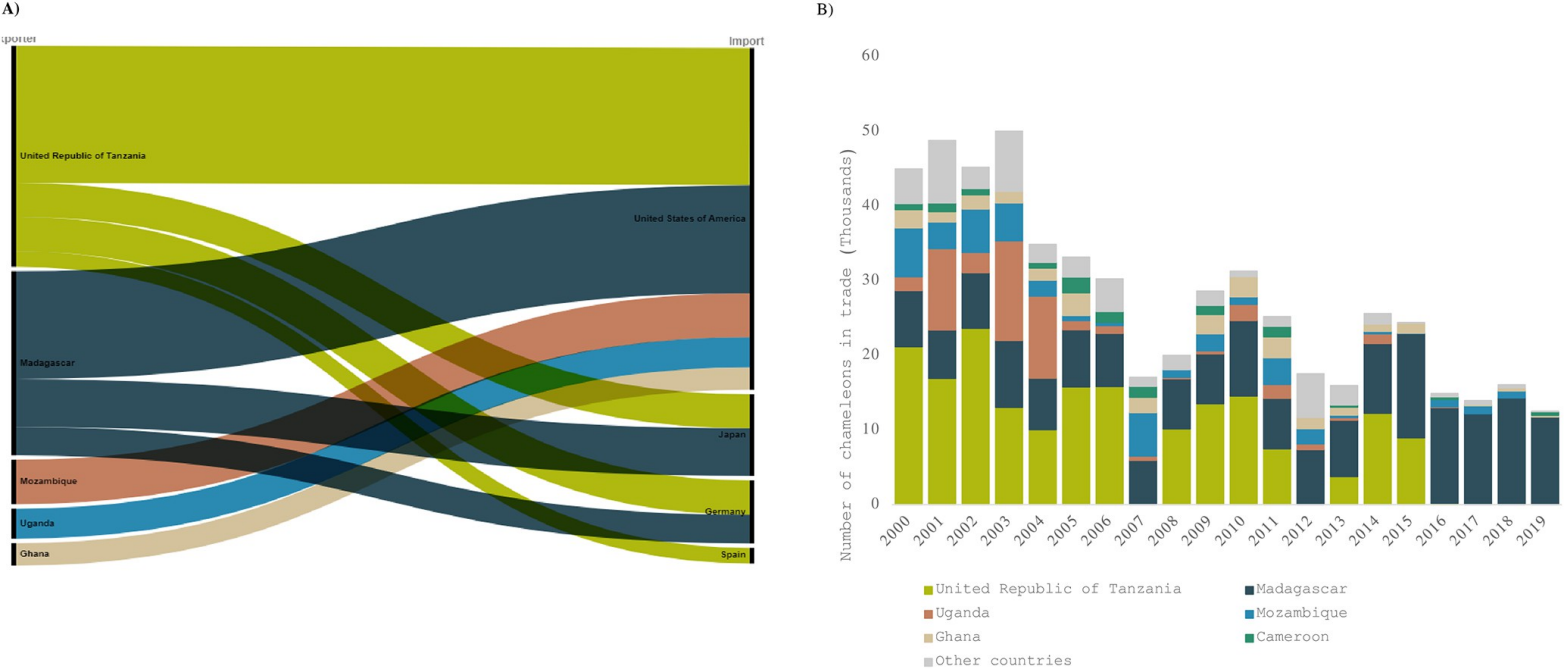
Geographical and temporal patterns in Chameleon trade. A) Main trade routes of live wild-sourced chameleons from African range States, 2000–2019, for commercial trade as reported by exporters. Country combinations represent 60% of total trade (327,552 individuals). Graph created in RawGraphs - https://app.rawgraphs.io/ - using Alluvial plot. B) Top six exporters of global direct live wild-sourced chameleon species, 2000–2019, for commercial trade as reported by exporters. Six countries trade represents 91% of total trade (498,734 individuals).

Top exporting countries for wild chamelons were within the species natural range, suggesting direct trade as opposed to a use of Hub markets (Fig 2). Tanzania was the largest exporter accounting for 34% of trade (187,367 chameleons). The USA dominated global chameleon imports accounting for 46% of trade (252,139 chameleons), with other importing nations including Japan (13%), and countries in Europe (especially Germany which alone accounted for 10% of global trade).

#### 3.1.1. Tanzania’s role in Chameleon trade

**Fig 3:**
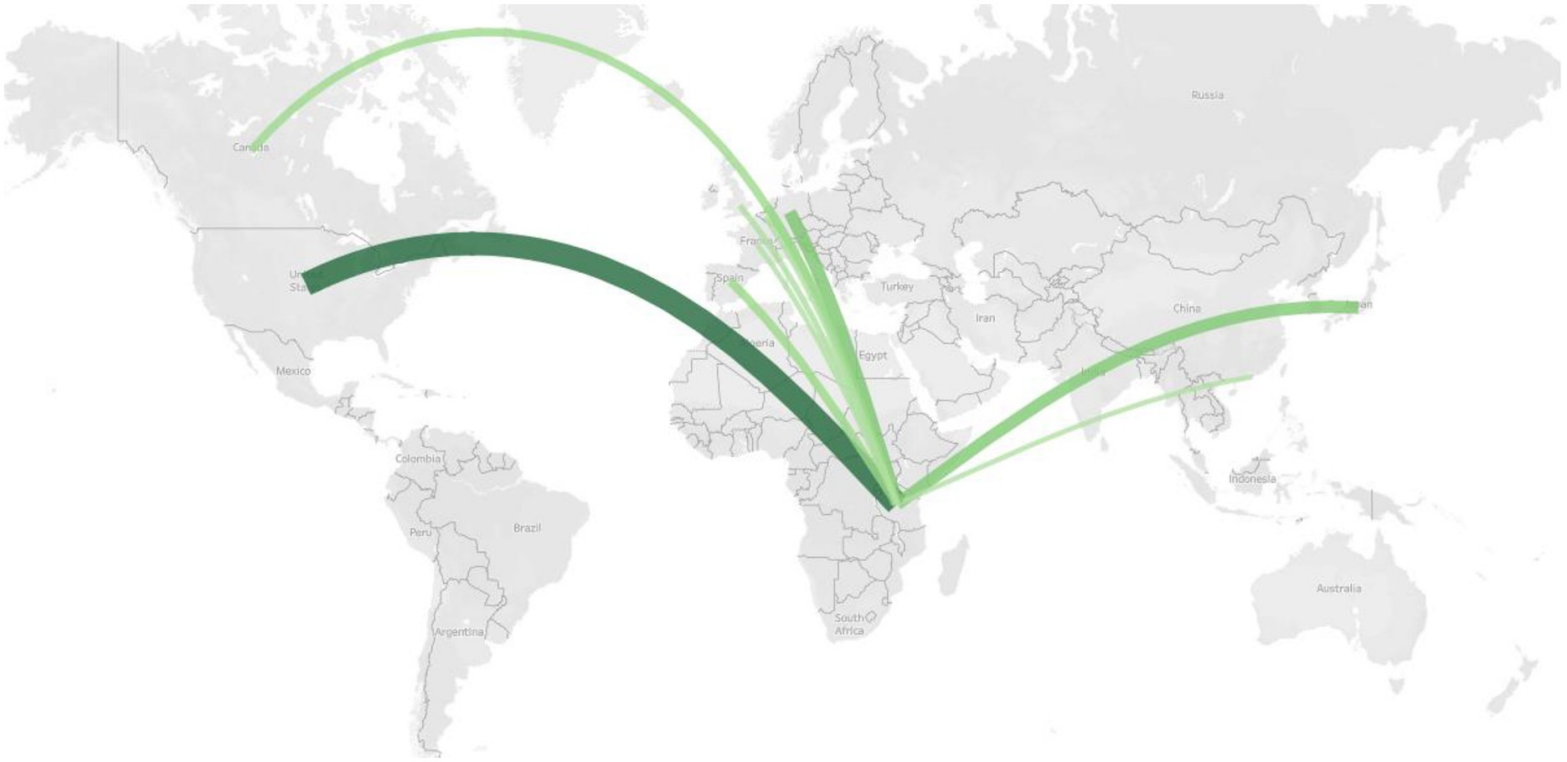
Map showing the destination of live, wild sourced Chameleon exports from Tanzania 2000–2019 (for graphical representation see S4 Fig in S1 Annex). All data were based on exporter-reported CITES trade data. (The designation employed and the presentation of material on this map do not imply the expression of any opinion whatsoever on the part of the Secretariat of the United Nations concerning the legal status of any country, territory, city or area or any of its authorities, or concerning the delimitation of its frontiers or boundaries).

With 34% of the global trade (187367 individual chameleons), Tanzania was the largest global exporter of chameleons over the study period. Tanzania exported primarily to the USA (48% of Tanzanian exports), western European countries such as Germany (12%) and Japan (12%) (Fig 3).

**Fig 4:**
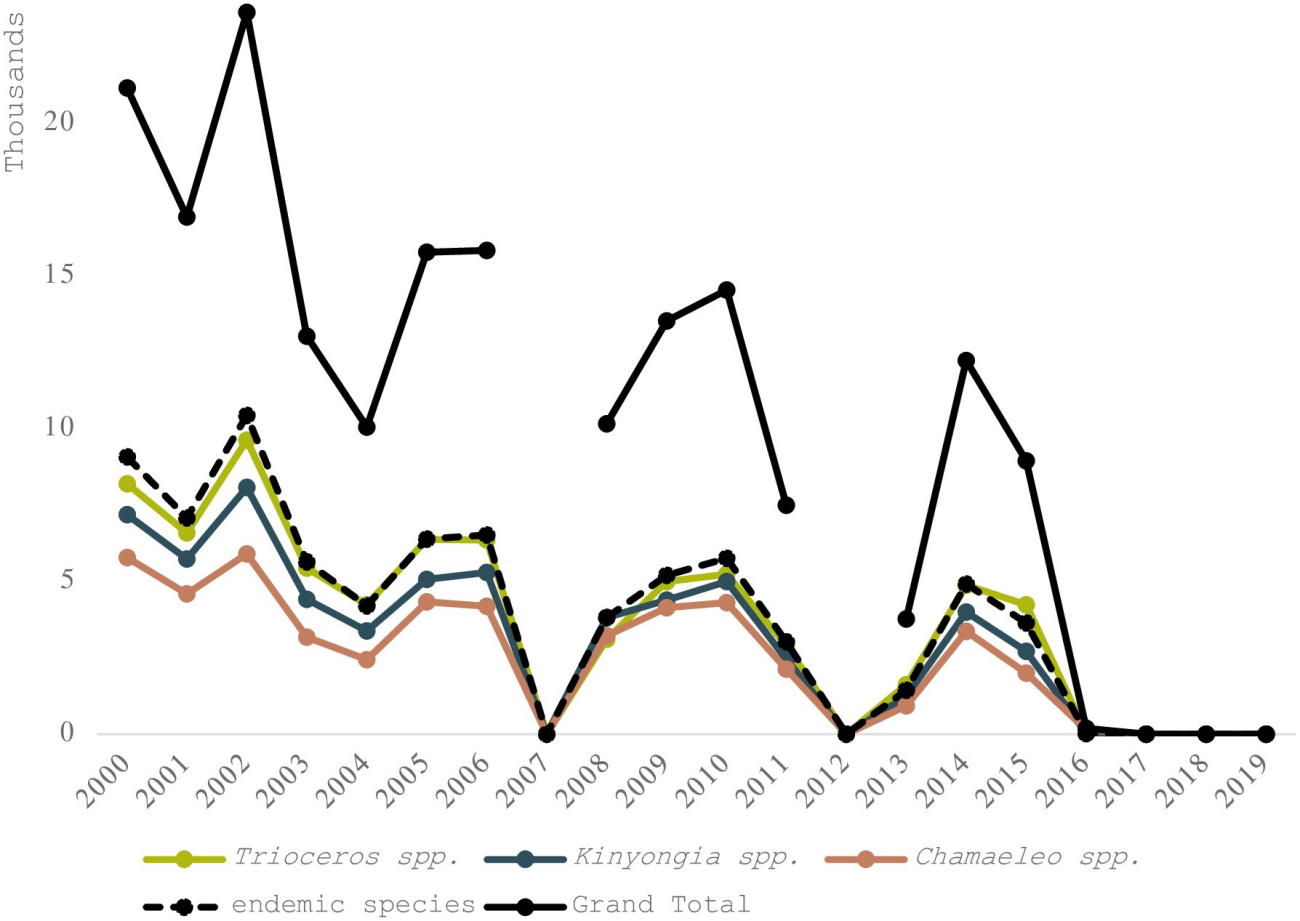
Direct exports of live, wild sourced Chameleons from Tanzania 2000–2019. Endemics comprise 41% of all trade in chameleons from Tanzania over the study period. Tanzania had not submitted their annual trade reports to CITES for years 2007 and 2012 at the time of data download. Species level graph detailed in S5a Fig in S1 Annex.

In contrast to global trends, trade from Tanzania is fairly evenly spread across the genera *Trioceros*, *Kinyongia* and *Chameleo* (Fig 4). Moreover, the export trend in Tanzanian endemic species, compared to non-endemic species, follows similar trends over time.

Some species appear to be more popular in trade than others and 6 species compromise 85% of trade (S5a Fig in S1 Annex). Looking more closely at Tanzanian endemic species we can see them clustering into three groups (S5b Fig in S1 Annex) The most traded cluster comprised *Kinyongia fischeri* and *K*. *tavetana*. The second cluster includes *Trioceros werneri*, *T*. *deremensis* and *T*. *fuelleborni* (until 2008). The least traded cluster contained *T*. *tempeli*, *K*. *uthmoelleri*, *K*. *oxyrhina and Rhampholeon spinosus* (S5b Fig in S1 Annex). Almost all trade in Tanzanian endemic species from *any source* were exported by Tanzania (S5b vs S5c Fig in S1 Annex): only 1.09% of exports in these species were from countries other than Tanzania and were all from captive-bred and ranched individuals (S3 Table in S1 Annex).

#### 3.1.2. Online trade of Tanzanian Chameleons

Of the 42 Tanzanian species assessed, there was evidence of online sale for 35(83.3%) species, and 29(69%) species were actively for sale with prices listed (species sold online detailed in S4b Table in S1 Annex). Prices were on average highest for *Trioceros* species, followed by *Kinyongia*, *Rieppeleon*, *Rhampholeon*, and *Chameleo* (S6 Fig in S1 Annex). There was no clear statistical relationship between price and endemism.

Our survey of nine hobbyist Facebook groups for chameleon suppliers and exotic pet stores provided little evidence that species are sold through Facebook as the respondents indicated that there is a strong awareness of the community guidelines which forbid the sale of live animals and this appears to be enforced stringently by curators of these pages for fear of having their accounts suspended. Largely these pages just existed as signposts for websites and events outside of Facebook selling these animals. There was also little evidence for sale of animals on Instagram although there are many active accounts posting photographs of their pet East African chameleons. The impact of such content on demand for these animals could be assessed using a more technology driven approach in future research (e.g. [24]).

The largest and most consistently updated online forum was found to be chameleonforums.com, run by long time hobbyists with knowledge of CITES regulations and the conservation status of many chameleons. However, there are many posts advertising shipments of endemic species, often priced only in private messages to the account posting the advert. This lack of transparency makes it hard to assess the volume and legality of trade in these species. Additionally, as was noted during surveying, there is a widespread belief amongst hobbyists that most species are threatened by habitat loss rather than collection for the pet trade.

#### 3.1.3. Collection of Chameleons in Tanzania

Our case study of interview responses from villagers, collectors and agents responsible for the first purchase of chameleons in Tanzania revealed that only two of the three mountain blocks surveyed had been participating in the chameleon trade, the East Usambara and the Uluguru. In both mountain ranges all responses related to trade in the past, with respondents agreeing that trade was no longer occurring, although estimates of how recent the trade was still functioning varied widely from a year to four or five years previously. Many villagers were aware that the trade was now banned by the government (a temporary ban on the export of all live wild animals was imposed in 2016, extended indefinitely in early February 2019).

Villagers mentioned higher rates of past trade from the Uluguru mountains, ~15,000 chameleons per annum, compared to ~6,000 in the East Usambara. These are conservative estimates as we only visited some villages, but the relative scales are perhaps comparable as similar numbers of villages were surveyed in each location (S7a Fig in S1 Annex).

More respondents in the Uluguru villagers were able to comment on the intricacies of the trade, however those in East Usambara reported higher prices per chameleon (4,475 Tanzania Shillings [1.95USD, January 2019] compared with 1,205 Tanzania Shillings [0.52USD]) (S7b Fig in S1 Annex). It was also clear from interviews that the presence of horns was a sought-after characteristic by traders, reflected in higher prices quoted for horned specimens (S7b and S7c Fig in S1 Annex).

Of the potential 13 species of chameleon that villagers reported historically harvesting and trading domestically for eventual export, 54% (7 species) were not reported in Tanzania’s official export data reported to CITES (Table 1). Amongst the perceived “high value” species, no exports of two-horned chameleons and 27 individuals from one-horned species (*Kinyongia oxyrhina*, *K*. *tenius & Rhampholeon spinosus*) present in either mountain range were reportedly exported by Tanzania during 2000–2019, this contrasts sharply with local estimates of “thousands” of these species collected annually (S7a Fig in S1 Annex).

## 4. Discussion

### 4.1. What do our findings and historical knowledge on Chameleon trade suggest for the future?

Our analysis shows an increase in ranching and captive breeding in chameleons, however this trend was largely driven by the export of widespread generalist species from countries outside of the species’ native ranges; the species involved did not change over the study period (S2c Fig in S1 Annex). Ranching or captive-breeding may provide a solution to meeting the demand for rarer, range restricted species, however more research would be needed to understand whether there are risks of this driving up demand and having potential perverse impacts on wild populations and local livelihoods.

In line with previous studies [1], our results show that countries with more diverse chameleon assemblages exported a greater number of live individual chameleons, and that a country’s chameleon species richness correlated with how species rich their chameleon trade was (S3a and S3b Fig in S1 Annex). While national legislation and trade quotas helped regulate exports from dominant countries rich in chameleon diversity, such as Kenya and Madagascar, this appeared to result in an increase in the number of countries trading in chameleons (1). The last UNEP-WCMC report on *Calumma* and *Furcifer* species found low levels of export following a removal of long-standing trade suspensions however noted that sourcing of the animals was still poorly understood and required surveying by independent investigators to avoid increased trade from wild-sourced populations or illegal trade [25]. However, we found both a decreasing volume of chameleon exports globally and a decreasing number of exporting countries across our study period of 2000–2019 (S3c Fig in S1 Annex) although we also showed an increase in the number of countries importing chameleons. This shrinking of the market (both in trade volume and sources) since Carpenter’s review [1], could possibly be due to more CITES regulations. It appears that these increased regulations and the resulting decrease in trade from historically important source countries Madagascar, Kenya and Tanzania has in fact not just driven trade elsewhere, at least when viewing CITES trade. This suggests that proper globally co-ordinated regulation in source countries can decrease global trade if done correctly.

Whilst the legal reported trade data provide insight into international demand and harvesting pressure, additional information on harvest/transit mortality, levels of illegal trade and better census data for the species’ wild populations is required to accurately assess the sustainability of harvesting chameleons for international trade.

### 4.2. How does the trading system work in Tanzania?

The structure of the supply chain in Tanzania appears similar to that identified in Madagascar; using casual local collectors, intermediaries and exporters [9]. Work in Madagascar also demonstrated the challenges of creating a sustainable trade in chameleons, and shifting profits from intermediaries to collectors, and addressing issues of illegal markets [26]. Whilst it is not clear if the same issues apply to the supply chains in Tanzania, there may transferable solutions or lessons learnt from Madagascar that could be applied in this situation.

Previous research identified that Usambara chameleons pass through a succession of intermediaries before export, but the pathways were not fully identified [10]. From the local interviews in the Uluguru and Usambara mountains it was clear that a fairly ordered system exists, connecting these remote mountains to the international market. Villagers in both mountain blocks mentioned agents coming from larger settlements at the base of the mountains (Muheza in the Usambara and Morogoro in the Uluguru) with an order for a certain quantity of chameleons with types specified (one horn, two horns, three horns, giant etc). The agents would give a time frame, usually a couple of days before returning to collect them or having them sent down the mountain with a courier, from there they would be transported to Dar es Salaam (although there was one account from the Usambara of chameleons historically being sent to Mombasa in neighboring Kenya). This broad structure of the supply network and trade routes was consistently given by the interviewees and although small differences were provided, such as greater or lesser amounts of known intermediary traders, the pattern of organized and specific orders was the same and opportunistic collecting was unheard of.

In the East Usambara this study was also able to contact and interview an agent operating between the mountain villages and the larger town of Muheza at the base of the mountain, an informal interview was conducted giving an insight into the chain of trade emanating from these mountains. He suggested that the speed of sourcing chameleons was more important than the number collected, suggesting that the market is top down controlled by demand, that permits are an afterthought and that these animals are wanted alive. He also mentioned targeting specific villages based on the chameleons needed to complete the order, with different altitudes and habitats yielding different species. His role as an intermediary was passed down to him from his father and he suggested there was a history of this trade in the region. As with most other people collecting in the region he had no interest in what the chameleons were needed for and was motivated by the income provided by them. His commentary on the value chain was speculative with him making only marginally more (~1000 Tanzania Shillings—$0.4 USD) than those collecting them for him in the villages and his perception being he received 30% of the value of the chameleons with those in Dar es Salaam making 70% more per chameleon. His commentary on the legality of the collection was perhaps the most telling, he was told by the agent in Muheza that they had permits for collection, however he was never shown these permits, additionally he was told to collect more than the permits allowed.

Across both mountain blocks awareness of why these chameleons were in demand was low, with the majority of respondents saying they did not know and/or that they did not care what the chameleons were used for after leaving the mountains, this reflected a view of wildlife products like these as being a source of income. In the Uluguru people were more aware of potential uses with many suggesting they were ‘ornamental’ and for ‘tourism’ with some suggestions being their use in snake parks and other attractions, this may explain some responses mentioning zoos and researchers sourcing from the mountains, a story also heard in the Usambara. However the majority of the respondents believed the chameleons collected left the country. Additionally one respondent mentioned the possibility they could be used for medicine and the general lack of medicinal uses was corroborated by a specialist in traditional medicine at Muhimbili University in Dar es Salaam; he said that in his years of research he had never seen or heard of chameleons being used in traditional medicine in Tanzania and found it unlikely to be an unknown market due to chameleons not being well liked in Tanzania and generally being seen as a bad omen.

### 4.3. Does Chameleon trade in Tanzania still continue?

At the time of the field work Tanzania had a ban on the export of live wild-sourced animals. Representatives from the Tanzania ports authority stated that large numbers of chameleons could not be leaving Tanzania through the airport or sea port in Dar es Salaam or through Mwanza on Lake Victoria due to regular checking of exports. However due to different management authorities on Zanzibar they could not comment on whether Zanzibar was being used as an exit point for live animals. Other experts (academic researchers and NGO staff) suggested that land borders with Kenya are easy to cross for Tanzanian people and there was evidence of trade occurring for rare timbers between the two countries (personal communication, August 2019). However, no evidence for chameleon trade via Zanzibar or Kenya was found during this study. Villagers interviewed in the Uluguru and Usambara mountains were also aware of a ban in wildlife trading, in many cases not having seen the agents in over a year. However, the length of time respondents had been aware of the ban varied widely from more than 5 years to around a year since last collecting.

This study found scant evidence for a chameleon trade from the Udzungwa mountains and the Magombera forest with only one respondent across both locations reporting being paid for finding chameleons. The Udzungwa mountain block is largely covered by national parks and nature reserves, with the highest protection afforded to areas close to major roads. From the interviews and follow up questions it seems that the national park regulations are well known of and strictly followed and travel to other sites proves to be unprofitable as villages surveyed around Udzungwa scarp nature reserve also had no record of chameleon trade.

Since carrying out this research, seizures of live Tanzanian chameleons being smuggled into Europe in 2021 does, unfortunately, indicate that illegal trade continues [27].

### 4.4. Caveats around the data collected in the field and possible sources of error

Interviews implied local sentiment towards conservation and the inherent value of nature in these mountain blocks may also have significant influences on the number, honesty and type of responses given. For example, the respondents in the Usambara were more hesitant to admit knowledge of the trade and reluctant to say selling these animals for profit was a good thing and expressed reservations about losing chameleons from the area; by contrast it appears that the residents of the Uluguru mountains did not mention these factors.

Another factor impacting the responses given in each mountain may be the longer conservation presence in the East Usambara with the establishment of Amani nature reserve, predating the Uluguru nature reserve by thirteen years (1997 vs 2009). This may be having a positive impact on local views towards conservation, however it may also have resulted in local people being less open to interviews on wildlife trade. It was infeasible within the scope of this study to establish the effect of this factor.

There are notable differences in the response quantity and quality over the two mountain blocks. In the Usambara there were fewer people able to comment on the trade compared to the Uluguru, however, reliability of the accounts received in the Usambara was higher due to many of the interviewees being people directly part of the collection and with first-hand knowledge of selling chameleons. Usambara villages were also closer together, with respondents reporting the same agents moving between villages, increasing the likelihood of repeated reports of harvesting incidents.

## Supporting information

S1 Annex Status and trends in the international wildlife trade in Chameleons from Tanzania. Supporting document containing supplementary tables and figures.
(DOCX)
