# Supplementary material for: Status and trends in the international wildlife trade in Chameleons with a focus on Tanzania
Source: PLoS One. 2024 May 16;19(5):e0300371. doi: 10.1371/journal.pone.0300371 (PMC11098367; doi:10.1371/journal.pone.0300371)
Supplement: S1 Annex — Supporting document containing supplementary tables and figures. (DOCX) [file pone.0300371.s001.docx]

# **Annex: Status and trends in the international wildlife trade in Chameleons from Tanzania**

Maxim Conrad Isaac^1^, Neil D. Burgess^1,2^, Oliver Tallowin^2^, Alyson Pavitt^2^, Reuben Kadigi^3^

*^1^ CMEC, within GLOBE institute, Universitetsparken 15, University of Copenhagen, DK-2100, Copenhagen, Denmark*

*^2^UN Environment Programme World Conservation Monitoring Centre (UNEP-WCMC), CB3 0DL, Cambridge, United Kingdom*

*^3^ Department of Food and Resource Economics, School of Agricultural Economics and Business Studies, Sokoine University of Agriculture, Morogoro, Tanzania*

Corresponding author: Maxim Conrad Isaac, Sortedam dossering 81, KBH Ø, 2100, Denmark, maxim.conrad.isaac@gmail.com ,(+45)91922360

Supplementary table 1. Manipulation of data, filters applied etc

| Preparing data | Filter data on unit codes so that only ‘blank’ is selected, this equates to only whole animal records |
| --- | --- |
|  | Excludes records with ‘purpose’ codes other than ‘T’ for commercial trade, other purposes deemed inconsequential to an analysis of global trade (see supplementary figure 1) |
|  | Set ‘source’ codes to all |
|  | Set values to ‘importer reported converted’ |
| Global trade | For global trade analysis exporter set to ‘all’ |
|  | For analysis of global market by genus (figure 2) data filtered by genus ‘all’, year filtered 2000-2016 (2017 data incomplete) |
|  | For analysis by exporter (figure 3) data was sorted by total exports by nation during the period 2000-2016 and the ten most prolific exporters were plotted over time in figure 3 |
|  | For analysis of sourcing in global chameleon trade (figure 4) data was analysed by source codes, sourcing analysis was conducted for the top ten exporting nations in two categories, range countries and non-range countries for chameleons, these were determined using the IUCN webpage (https://www.iucnredlist.org) |
|  | For analysis of trade diversity (Supplementary figure 2) trade records were ordered by taxon and then using the COUNTIF function were used to create a binary count of whether each species in trade during the study period was in trade each year. This generated a species count for each year, plotted in Supplementary figure 2a.This was then plotted against export numbers (supplementary figure 2b) and additionally plotted against the number of chameleon species present in each nation (supplementary figure 2c) |
| Trade from Tanzania | Filter data by exporter, exporter defined to only ‘United Republic of Tanzania’ |
|  | ‘importer’ field set to ‘all’ and plotted over the study period (supplementary figure 3) to provide insights into export market |
|  | To assess species profile of trade data refined by ‘genus’ |
|  | Trade in endemics assessed by refining data by taxon using Species + data to determine Eastern Arc mountain range endemics (see supplementary table 2) endemic trade shown in supplementary figure 4 |
|  | To compare in the field findings to CITES trade database records exports from Tanzania were limited to those species present in survey areas (detailed in supplementary table 2) to create figure (X) |

Supplementary table 2: EAM endemic species found in CITES trade database *indicated newly described species not yet evaluated by the reptile database and so not included in the analysis of chameleon trade using the CITES data

| **Species** | **Threat assessment** | **trend** | **Estimated area of occupancy (KM^2^)** | **Approximate range** | **Present in CITES trade database 2000-2016** | **Present in survey areas** | **Range countries** | **Tanzanian endemic** |
| --- | --- | --- | --- | --- | --- | --- | --- | --- |
| *Kinyongia fischeri* | NT | Unknown | 248 | Nguru and Nguu Mountains | yes | no | Tanzania | Yes |
| *Kinyongia magomberae* | EN | Unknown | 105 | Magombera forest | no | yes | Tanzania | Yes |
| *Kinyongia matschiei* | EN | Decreasing | 288 | East Usambara | no | yes | Tanzania | Yes |
| *Kinyongia msuyae* | Data deficient | Data deficient | Data deficient | Livingstone mountains | no | no | Tanzania | Yes |
| *Kinyongia multituberculata* | EN | Decreasing | 354 | East and West Usambara mountains | no | yes | Tanzania | Yes |
| *Kinyongia oxyrhina* | NT | Stable | 1474 | Uluguru, Udzungwa, Nguru and Rubeho Mountains | yes | yes | Tanzania | Yes |
| *Kinyongia tavetana* | NT | Decreasing | 1267 | Kilimanjaro, Mt. Meru, North Pare Mountains, South Pare Mountains. Chyulu Hills (Kenya) | yes | no | Tanzania, kenya | No |
| *Kinyongia uluguruensis* | LC | Stable | 264 | Uluguru mountains | no | yes | Tanzania | Yes |
| *Kinyongia uthmoelleri* | LC | Stable | 3730 | Mount Hanang, the Ngorongoro crater highlands (including Mt. Oldeani) and the South Pare Mountains | yes | no | Tanzania | Yes |
| *Kinyongia vanheygeni* | LC | Decreasing | 274 | Poroto and Rungwe Mountains | no | no | Tanzania | Yes |
| *Kinyongia vosseleri* | EN | Decreasing | 288 | East Usambara mountains | no | yes | Tanzania | Yes |
| *Rhampholeon acuminatus* | CR | Unknown | 28 | Nguru Mountains | no | no | Tanzania | Yes |
| *Rhampholeon beraduccii* | VU | Unknown | 14 | Mahenge Mountains | no | no | Tanzania | Yes |
| *Rhampholeon colemani** | Data deficient | Unknown | Data deficient | Uzungwa Scarp Nature Reserve | no | yes | Tanzania | Yes |
| *Rhampholeon moyeri* | LC | Stable | 1590 | Udzungwa mountains | no | yes | Tanzania | Yes |
| *Rhampholeon nicolai** | Data deficient | Unknown | Data deficient | Ukaguru mountains | no | no | Tanzania | Yes |
| Rhampholeon princeeai * | Data deficient | unknown | Data deficient | Nguru Mountains | no | no | Tanzania | Yes |
| *Rhampholeon rubeho** | Data deficient | Unknown | Data deficient | Rubeho Mountains | no | no | Tanzania | Yes |
| *Rhampholeon sabini** | Data deficient | Unknown | Data deficient | Nguu North and Kilindi Forest | no | no | Tanzania | Yes |
| *Rhampholeon spinosus* | EN | Decreasing | 567 | East and West Usambara Mountains | yes | yes | Tanzania | Yes |
| *Rhampholeon temporalis* | EN | Decreasing | 288 | Usambara mountains | no | yes | Tanzania | Yes |
| *Rhampholeon uluguruensis* | LC | Stable | 278 | Uluguru Mountains | no | yes | Tanzania | Yes |
| *Rhampholeon viridis* | EN | Decreasing | 152 | North and South Pare Mountains | no | no | Tanzania | Yes |
| *Rhampholeon waynelotteri** | Data deficient | Unknown | Data deficient | Kanga and Nguru mountains | no | no | Tanzania | Yes |
| *Trioceros deremensis* | LC | Stable | 1400 | East Usambara, Uluguru, Nguu and Nguru Mountains | yes | yes | Tanzania | Yes |
| *Trioceros fuelleborni* | LC | Stable | 415 | Poroto Mountains and northern volcanoes of the Rungwe volcanic complex | yes | no | Tanzania | Yes |
| *Trioceros hanangensis* | NT | Unknown | 15 | Mt. Hanang | no | no | Tanzania | Yes |
| *Trioceros incornutus* | LC | Stable | Unknown | Poroto, Rungwe and Ukinga Mountains |  | no | Tanzania, Malawi | No |
| *Trioceros laterispinis* | EN | Decreasing | 538 | Udzungwa Mountains | yes | yes | Tanzania | Yes |
| *Trioceros sternfeldi* | LC | Stable | 3660 | Mt. Meru, Mt. Kilimanjaro, and the Ngorongoro crater highlands | yes | no | Tanzania | Yes |
| *Trioceros tempeli* | LC | Stable | 5000 | Udzungwa Mountains | yes | no | Tanzania | Yes |
| *Trioceros werneri* | LC | Stable | 5500 | Udzungwa, Uluguru, Ukaguru, Nguru and Rubeho Mountains | yes | yes | Tanzania | Yes |

|  | | Taxon | | | | |
| --- | --- | --- | --- | --- | --- | --- |
| **Source** | **Exporter** | ***Kinyongia fischeri*** | ***Trioceros deremensis*** | ***Trioceros laterispinis*** | ***Trioceros werneri*** | **Grand Total** |
| **Captive bred** | United States of America |  |  |  |  | 35 |
|  | Canada |  | 6 |  | 15 | 21 |
|  | Belgium |  |  | 16 |  | 16 |
| **Born in captivity** | Kenya | 675 |  |  |  | 675 |
| **Ranched** | Kenya | 40 |  |  |  | 40 |
| **Wild caught** | Democratic Republic of the Congo |  | 100 |  |  | 100 |
| **Grand Total** |  | **715** | **106** | **16** | **15** | **887** |

Supplementary table 3: Exports of Tanzanian endemic chameleons from countries other than Tanzania globally and the sources of these exports. The wild caught record in the DRC would represent a significant range expansion and is considered a misclassified record. This trade is representative of 1.09% of trade in these species

Supplementary table 4a: most common Online marketplaces for Tanzanian species

| **Website** | **Number of species for which it appeared in search results** |
| --- | --- |
| **chameleonforums.com** | **29** |
| **reptileforums.co.uk** | **18** |
| **Flchams.com** | **13** |
| **exotic-pets.co.uk** | **13** |
| **Backwaterreptiles.com** | **10** |
| **lllreptile.com** | **8** |
| **faunaclassifieds.com** | **6** |
| **snakesatsunset.com** | **4** |
| **undergroundreptiles.com** | **4** |
| **speciestrader.com** | **4** |
| reptmart.com | 3 |
| reptiles4all.com | 2 |
| cbreptile.com | 2 |
| reptilesncritters.com | 1 |
| gotreptiles.com | 1 |
| reptilecity.com | 1 |
| freshmarine.com | 1 |
| reptilerapture.net | 1 |

Supplementary table 4b: Online marketplaces surveyed and species presence for sale on each

| **Chameleon species** | **Online marketplace** | | | | | | | | | | | | | | | |
| --- | --- | --- | --- | --- | --- | --- | --- | --- | --- | --- | --- | --- | --- | --- | --- | --- |
|  | **snakesatsunset.com** | **backwaterreptiles.com** | | **flchams.com** | | **undergroundreptiles.com** | **chameleon forums** | | **reptileforums.co.uk** | **speciestrader.com** | | [**faunaclassifieds.com**](http://www.faunaclassifieds.com/) | | [**exotic-pets.co.uk**](https://www.exotic-pets.co.uk/) | **lllreptile.com** |  |
| *Kinyongia fischeri* | x |  | |  | |  | x | | x | x | | x | | x | x |  |
| *Kinyongia magomberae* |  |  | |  | |  |  | |  |  | |  | |  |  |  |
| *Kinyongia matschiei* |  | x | | x | |  | x | |  |  | |  | |  |  |  |
| *Kinyongia multituberculata* |  | x | | x | |  | x | | x |  | | x | |  |  |  |
| *Kinyongia oxyrhina* |  |  | |  | |  |  | |  |  | | x | |  |  |  |
| *Kinyongia tavetana* |  | x | |  | |  | x | | x | x | | x | |  |  |  |
| *Kinyongia uluguruensis* |  |  | |  | |  | x | |  | x | |  | |  |  |  |
| *Kinyongia uthmoelleri* |  |  | |  | |  | x | | x |  | | x | |  |  |  |
| *Kinyongia vanheygeni* |  |  | |  | |  |  | |  |  | |  | |  |  |  |
| *Kinyongia vosseleri* |  |  | |  | |  |  | |  |  | |  | |  |  |  |
| *Rhampholeon acuminatus* |  | | assorted | x |  | | x | x | | | x | x | x | |  |  |
| *Rhampholeon beraduccii* |  | |  |  |  | |  |  | | |  |  |  | |  |  |
| *Rhampholeon moyeri* |  | |  |  |  | |  |  | | |  |  |  | |  |  |
| *Rhampholeon spinosus* |  | |  |  |  | | x | x | | |  |  |  | |  |  |
| *Rhampholeon temporalis* |  | |  | x |  | | x | x | | | x | x | x | |  |  |
| *Rhampholeon uluguruensis* |  | |  |  |  | |  | x | | | x | x | x | |  |  |
| *Rhampholeon viridis* |  | |  |  |  | | x | x | | | x | x | x | |  |  |
| *Rieppeleon brevicaudatus* |  | |  | x | x | | x | x | | | x | x | x | | x |  |
| *Trioceros deremensis* | x | | x | x |  | | x | x | | | x |  |  | | x |  |
| *Trioceros fuelleborni* |  | |  | x |  | | x | x | | |  |  |  | |  |  |
| *Trioceros hanangensis* |  | |  |  |  | | x | x | | |  |  |  | |  |  |
| *Trioceros laterispinis* |  | |  |  |  | | x | x | | |  |  |  | |  |  |
| *Trioceros sternfeldi* |  | |  | x |  | | x | x | | | x |  |  | | x |  |
| *Trioceros tempeli* |  | |  |  |  | | x |  | | |  | x |  | |  |  |
| *Trioceros werneri* | x | | x | x |  | | x | x | | |  | x |  | | x |  |
| *Chamaeleo anchietae* |  | |  |  |  | |  |  | | |  |  |  | |  |  |
| *Chamaeleo dilepis* |  | | x | x | x | | x | x | | | x |  | x | | x |  |
| *Chamaeleo gracilis* |  | | x |  | x | | x | x | | |  | x |  | | x |  |
| *Chamaeleo laevigatus* |  | |  |  |  | |  |  | | |  |  |  | |  |  |
| *Rhampholeon boulengeri* |  | |  |  |  | |  |  | | |  |  |  | |  |  |
| *Rhampholeon nchisiensis* |  | |  |  |  | |  | x | | | x | x | x | |  |  |
| *Rieppeleon brachyurus* |  | |  |  |  | |  | x | | | x |  |  | |  |  |
| *Rieppeleon kerstenii* |  | |  |  |  | |  | x | | | x |  |  | |  |  |
| *Trioceros bitaeniatus* |  | | x | x |  | | x | x | | | x | x | x | | x |  |
| *Trioceros ellioti* | x | | x |  | x | | x | x | | |  | x | x | | x |  |
| *Trioceros incornutus* |  | |  |  |  | | x |  | | |  |  |  | |  |  |
| *Trioceros jacksonii* | x | | x | x | x | | x | x | | | x | x | x | | x |  |
| *Trioceros melleri* | x | | x | x | x | | x | x | | | x | x | x | | x |  |
| *Trioceros rudis* |  | | x | x | x | | x | x | | | x | x | x | | x |  |
| *Kinyongia tenuis* |  | |  |  |  | | x |  | | |  |  |  | |  |  |
| *Trioceros goetzei* |  | |  |  |  | |  |  | | |  |  |  | |  |  |

Supplementary table 5: questions and prompts used in the semi structured interviews conducted in the eastern arc mountains of Tanzania

| Standard interview questions:   - Are you aware of people selling chameleons around here? - How often do you/they go and collect chameleons? - How many do you/they collect? - Are there particular types of chameleon you/they collect? - Did you/they used to collect chameleons more or less than you do now? - Are certain chameleons easier or harder to find now? - Who do you/they sell the chameleons to? - How much money do you/they get paid per chameleon? - Do they buyers tell local people why they want the chameleons? - Do you know anything about farming/ranching chameleons? - To your knowledge is collecting chameleons legal or illegal? |
| --- |
| In areas where the respondents had never heard of chameleon trade more broad questions were asked:   - Have you ever heard of or are interested in chameleon farming? - To your knowledge is trading animals like chameleons legal or illegal? - Is it possible people are collecting animals from inside the national park/nature reserve? - Do you see chameleons more or less than you used to? - Why do you think there are more/less chameleons? - What kind of chameleons do you see? - What is your opinion of chameleons - Are other animals collected in this area?   - (if so) Who are they bought by?   - How often do buyers come?   - Do the buyers tell you why they want them?   - When did they last come here?   Additionally these questions were occasionally asked as follow up questions to villagers that did know of the chameleon trade. |


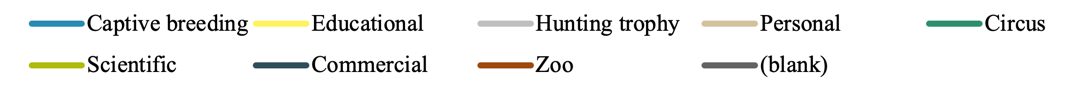


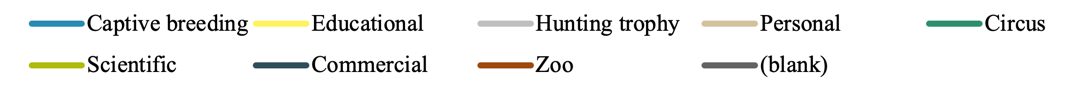


Supplementary figure 1: Purpose of exports in global chameleon trade. A) changes in purpose of exports over time. B)Total exports by purpose over the study period


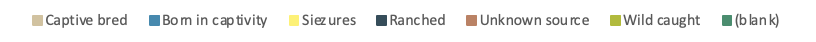


Supplementary figure 2a: sourcing of chameleon exports by country of origin and by genus

Supplementary figure 2b: Species diversity of various sourcing methods globally 2000-2019

Supplementary figure 2c: Global trade in *Chamaeleonidae* 2000-2019 divided by genus

Supplementary figure 3a: Trade diversity across the study period

Supplementary figure 3b: Trade diversity in relation to species richness of the trading nation 2000-2019

Supplementary figure 3c: Diversity of nations participating in global trade

Supplementary figure 4: Destination of Tanzanian chameleon exports 2000-2016, this is a graphical representation of figure 4.

Supplementary figure 5a: Direct exports from Tanzania of live, wild-sourced chameleon genera, 2000-2019, for commerical trade as reported by Tanzania. six chameleon genera represent 85% of total (514,597 individuals).

Supplementary figure 5b: Tanzanian exports of Tanzanian endemic species **K. tavetana* also documented as possibly present in southern kenya EAM

Supplementary figure 5c: Global trade in Tanzanian endemic chameleons

Supplementary figure 6: A graph showing the average prices charged online for chameleon species found in Tanzania, Tanzanian endemics are in block colours, non-endemics are lightly shaded. Colours represent different genera

Supplementary figure 7a: Graphs showing the average yearly exports from each village sampled in the Uluguru and Usambara. Standard deviation was high in the Uluguru but for the Usambara was not possible to plot due to the small number of interviewees able to estimate numbers with confidence. Number of chameleons collected yearly

Supplementary figure 7b: Average prices paid to local collectors in Tanzanian shillings (1USD= 2299TSH [01/08/2019]) for different chameleon categories. In the Uluguru the two horned species commanded the highest prices, whilst in the Usambara it was three horned species. Across both mountain ranges the lowest value chameleons were those without horns. Prices were consistently much higher in the Usambara compared to the Uluguru mountains as shown by the average columns of 4,475TSH compared with 1,205TSH. Giant chameleons were excluded from this graph for clarity due to the fact that the prices quoted were very high and from limited sources, casting doubt on their reliability


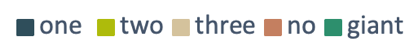


Supplementary figure 7c: Pie charts showing the showing the percentage of interviewees that mentioned each category of chameleon as being sought by collectors in the Usambara and Uluguru mountains and how this is divided across the numbers quoted by interviewees

Supplementary figure 8: differences between importer and exporter reporting for chameleon trade from Tanzania

|  | | | | | |
| --- | --- | --- | --- | --- | --- |
|  | **One horn** | **Two horns** | **Three horns** | **No horn** | **Giant** |
| Uluguru | *K. oxyrhina* | *k. uluguruensis* | *T. deremensis* | *Rieppeleon brachyurus* | *T. melleri* |
|  |  |  | *T. werneri* | All *Chameleo* species |  |
|  |  |  |  | females of species |  |
| Usambara | *K. tenuis* | *K. matschiei* | *T. deremensis* | *“* | *T. melleri* |
|  |  | K. vosseleri |  |  |  |
|  | *Rhampheleon spinosus* | K. multiturbuculata |  |  |  |
|  | *Rhampheleon temporalis* |  |  |  |  |

Supplementary figure 9: Tanzanian exports of species present in survey areas, 2000-2019
